# Aptamer-Conjugated Multifunctional Polymeric Nanoparticles as Cancer-Targeted, MRI-Ultrasensitive Drug Delivery Systems for Treatment of Castration-Resistant Prostate Cancer

**DOI:** 10.1155/2020/9186583

**Published:** 2020-04-25

**Authors:** Youqiang Fang, Shaoxiong Lin, Fei Yang, Jie Situ, Shudong Lin, Yun Luo

**Affiliations:** ^1^Department of Urology, The Third Affiliated Hospital of Sun Yat-sen University, Guangzhou 510630, China; ^2^Department of Otorhinolaryngology, The First Affiliated Hospital of Shantou University Medical College, Shantou 515041, China; ^3^Guangzhou Institute of Chemistry, Chinese Academy of Sciences, Guangzhou 510650, China

## Abstract

Nanoscopic therapeutic systems that incorporate therapeutic agents, molecular targeting, and imaging capabilities have gained momentum and exhibited significant therapeutic potential. In this study, multifunctional polymeric nanoparticles with controlled drug delivery, cancer-targeted capability, and efficient magnetic resonance imaging (MRI) contrast characteristics were formulated and applied in the treatment of castration-resistant prostate cancer (CRPC). The “core-shell” targeted nanoparticles (NPs) were synthesized by the self-assembly of a prefunctionalized amphiphilic triblock copolymer composed of poly(lactic-co-glycolic-acid) (PLGA), polyethylene glycol (PEG), and the Wy5a aptamer (Apt), which have been screened for targeting the CRPC cell line PC-3 by cell-SELEX technique as described in our previous study. Docetaxel (Dtxl) and a cluster of hydrophobic superparamagnetic iron oxide (SPIO) nanoparticles were simultaneously encapsulated into the targeted nanoparticles. The targeted NPs showed a controlled drug release and an increased contrast-enhanced MRI capability. The presence of Wy5a on the nanoparticle surface resulted in the cancer-targeted delivery to PC-3 cells *in vitro* and *in vivo*. *In vitro* MRI and cytotoxicity studies demonstrated the ultrasensitive MRI and increased cytotoxicity of these targeted NPs. *In vivo* studies revealed that the targeted NPs exhibited a more efficacious antitumor capability without significant systemic toxicity. Our data suggested that these targeted NPs may be a promising drug delivery system for the efficacious treatment of CRPC.

## 1. Introduction

Despite the recent advances in prostate cancer therapy, such as improved surgical strategies and new drugs in androgen deprivation therapy, the clinical prognosis of patients diagnosed with castration-resistant prostate cancer (CRPC) remains poor [1, 2]. Docetaxel (Dtxl) has been widely used as the first-line chemotherapeutic drug for CRPC and showed a survival advantage [1]. However, the therapeutic efficacy of Dtxl can be compromised by its nonselective toxicity and propensity to induce drug resistance [3]. In addition, it is difficult to monitor the drug pharmacokinetic and therapeutic effect due to the lack of powerful imaging modalities, though magnetic resonance imaging (MRI) has witnessed one of the most powerful techniques in the field of prostate cancer imaging [4]. The improvement in multifunctional nanoparticle technology for combined targeted drug delivery and tumor imaging provides new opportunities to overcome these obstacles [5]. It has been reported that stable GO-coated Fe3O4 nanocomposites are successfully used as a robust drug delivery carrier for simultaneous tumor MR imaging and targeted therapy [5]. Moreover, the innovative contrast agents for tumor imaging such as ultrasmall bimetallic bovine serum albumin-directed gold-silver (Au-Ag@BSA) nanoparticles have attracted significant attention for potential applications in the fields of multifunctional nanomedicine [6].

Over the past two decades, polymeric nanoparticles based on biodegradable amphiphilic block copolymers have been emerging as a multifunctional nanoplatform for cancer targeting, drug delivery, and tumor imaging application with the advancement of polymer engineering [7, 8]. The amphiphilic copolymers can spontaneously assemble into nanoparticles in an aqueous environment. Moreover, multifunctional polymeric nanoparticles constructed by the combination of nanotechnology and polymer chemistry have shown significant therapeutic potential [9]. A central challenge is that the multifunctional polymeric nanoparticles should be conferred with the capacity of molecular targeting, immune evasion, and drug release to overcome the physiological barriers *in vivo*. The amphiphilic block copolymers usually contain the hydrophilic and hydrophobic segments. The hydrophilic segment renders the nanoparticles “stealth” by decreasing nonspecific immune clearance and biofouling by plasma protein, and the reactive groups for the conjugation of targeting ligands to direct the specific delivery of nanoparticles, whereas the hydrophobic segment is utilized as a controlled-release polymer system to load the chemotherapeutic drugs and MRI contrast agents [10]. Therefore, the polymeric nanoparticles formulated by amphiphilic block copolymers are characterized by a core-shell structure, in which the hydrophobic core is capable of carrying hydrophobic drugs, the hydrophilic shell provides the stealth and functional groups, and the targeting ligands are conjugated to the surface of nanoparticles.

Aptamer (Apt) has attracted extensive attention as targeting ligands, which are single-stranded oligonucleotides that fold into unique tertiary structures to bind to the targets. Aptamer has high affinity and specificity the same as antibodies. However, aptamer has multiple unique advantages, such as ease of chemical synthesis and modification, low-molecular weight, high stability, rapid tissue penetration, and lack of immunogenicity. In prostate cancer, A10 2′-fluoropyrimidine RNA aptamer binding to prostate-specific membrane antigen (PSMA) has been widely applied as the targeting ligands for targeted nanoparticles [11]. However, the expression of PSMA is relatively low in the cancer cells of CRPC. Therefore, in our previous study [12], a new aptamer, Wy5a, specially binding to the cancer cells of CRPC (PC-3) without prior knowledge of the exact membrane target protein, has been developed by the SELEX technology (Systematic Evolution of Ligands by Exponential Enrichment) using the living whole cells. In this study, the aptamer Wy5a was utilized as targeting ligands to verify its capability of targeting CRPC *in vitro* and *in vivo*.

In this study, multifunctional polymeric nanoparticles were applied in tumor-targeted drug release and MRI with a simple, efficient, and more controllable system using a well-defined and predictable formulation strategy. The nanoparticles were formulated with three biomaterials: poly(D,L-lactide-co-glycolide) (PLGA) was chosen for the hydrophobic core to load Dtxl and MRI contrast agent SPIO (approximately 6 nm), poly(ethylene glycol) (PEG) was selected as a hydrophilic shell to stabilize the nanoparticles and provide functional-end groups for the attachment of targeting ligands, and aptamer Wy5a was employed as the hydrophilic targeting ligands for cancer targeting. Unlike our previous protocols to formulate the nanoparticles [13], herein we developed the prefunctionalized amphiphilic triblock copolymer (PLGA-b-PEG-b-Apt) bearing all of the three desired NP components and formulated the targeted nanoparticles by macromolecular self-assembly in an aqueous environment in one single step. Aptamer surface density was easily regulated by using distinct ratios of PLGA-b-PEG-b-Apt triblock copolymer with PLGA-b-PEG diblock copolymer without the targeting aptamer to avoid the unnecessary masking of PEG on NP surface by excess aptamer, which could compromise the stealth property of PEG. Furthermore, the biophysicochemical properties of multifunctional nanoparticles were characterized, and the antitumor and differential targeting capabilities were assessed.

## 2. Results

### 2.1. Formulation of Targeted Nanoparticles

**Figure 1:**
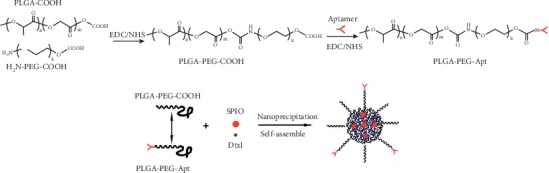
Synthetic scheme and preparation process for aptamer-conjugated multifunctional polymeric nanoparticles. PEG, poly(ethylene glycol); EDC, 1-ethyl-3-(3-dimethylaminopropyl)-carbodiimide; NHS, N-hydroxysuccinimide; PLGA, poly(D,L-lactic-co-glycolic acid); SPION, superparamagnetic iron oxide nanoparticles; Dtxl, docetaxel.

As shown in Figure 1, a nanoprecipitation technique was adopted to prepare the NPs. To avoid the incidence of postsynthesis NP surface modification, the prefunctionalized biointegrated block copolymer that contains all of the three desired NP components was firstly developed. The PLGA-b-PEG3.4k-b-Wy5a triblock copolymer was synthesized by a two-step reaction (Figure 1). Firstly, the amine terminal of heterobifunctional PEG (NH_2_-PEG-COOH) was conjugated to the carboxyl-functionalized PLGA (PLGA-COOH) using EDC/NHS as catalysts, resulting in PLGA-PEG-COOH diblock copolymer. Secondly, the amine functional group of Wy5a was conjugated to the carboxyl group of PLGA-PEG-COOH. The targeted nanoparticles were self-assembly formulated by precipitating the copolymers of PLGA-b-PEG-b-Wy5a/PLGA-PEG-COOH in water without postparticle modification. The targeted nanoparticles formed a core-shell structure in an aqueous solution. The hydrophobic PLGA block formed the core to provide a matrix for the encapsulation of Dtxl and hydrophobic SPIO, while the hydrophilic PEG formed a corona-like shell to stabilize the particles. The hydrophilic aptamer Wy5a, which was protruded from the surface of nanoparticles, provided the targeting capability. The Aptamer on the NP surface was determined by Apt cleavage assay and nucleic acid quantification after hydrolyzing the aptamer from the NP surface as described previously [14]. The quantity of Aptamer on the NP surface was calculated as 18.47 ± 0.93 *μ*g per mg of NPs.

### 2.2. Characterization of Targeted Nanoparticles

**Figure 2:**
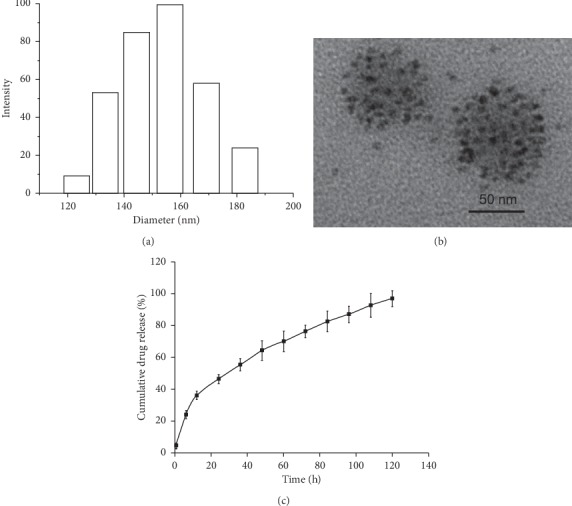
(a) Dynamic light-scattering histogram showing the size distribution of targeted nanoparticles. (b) Transmission electron microscopic images of Wy5a-SPIO/Dtxl-NPs. (c) Kinetics of physicochemical release showed the controlled release of Dtxl.

DLS was adopted to characterize the NP hydrodynamic size, polydispersity, and zeta potential. As shown in Figure 2(a), the average hydrodynamic size of the targeted nanoparticles was approximately 154.3 nm in a phosphate buffered saline. The average zeta potential of targeted nanoparticles was approximately −38.4 mv. The morphology of NPs was observed under TEM. TEM images revealed that the targeting nanoparticles Wy5a-SPIO/Dtxl-NPs showed a well-defined spherical shape, and a cluster of dispersive SPION was successfully encapsulated in the particles. Because PEG-PLGA copolymer and Dtxl did not significantly attenuate the electron beams under TEM, Wy5a-SPIO/Dtxl-NPs were mainly present as isolated clusters of SPIO nanoparticles. The anticancer drug Dtxl and MRI contrast agent SPIO were simultaneously encapsulated in the NPs at a relatively high loading content of 5.78 ± 0.83% (w/w) and 8.34 ± 1.42% (w/w), respectively. The *in vitro* drug release profile was shown in Figure 2(c). An initial burst of 36% accumulative release was observed in the first 12 h for Wy5a-SPIO/Dtxl-NPs, followed by a sustained drug release for another more than 108 h.

### 2.3. Magnetization Loops and MRI Phantom

**Figure 3:**
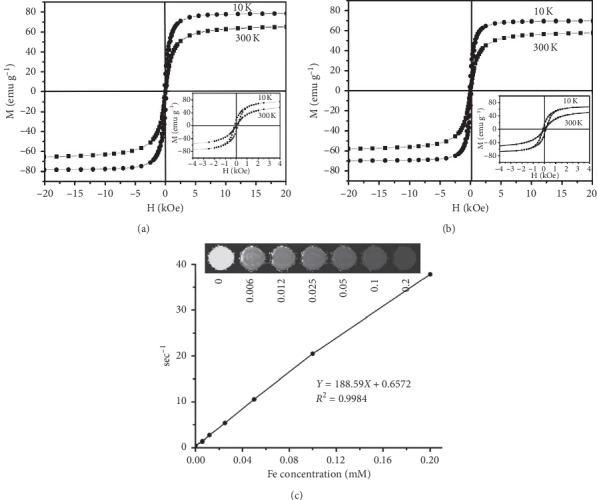
Hysteresis loops of 6 nm SPION (a) and Wy5a-SPIO/Dtxl-NPs (b) measured at 10 K and 300 K. The two insets in the figures showed the local magnification. (c) T2-weighted imaging of Wy5a-SPIO/Dtxl-NPs at the different iron concentration and T2 relaxation rate as a function of the iron concentration of Wy5a-SPIO/Dtxl-NPs.

As an MRI contrast agent, SPION can weaken the signal, by which the diseased tissues are differentiated from the surrounding normal tissues. To generate an ideal contrast in MRI signal strength, MRI contrast agents should be able to relax magnetic moment vectors rapidly when the applied magnetic field is removed. As shown in Figures 3(a) and 3(b), the magnetization loops of the hydrophobic SPION and Wy5a-SPIO/Dtxl-NPs were measured at both 10 K and 300 K at room temperature. Both the hydrophobic SPION and Wy5a-SPIO/Dtxl-NPs were ferromagnetic at 10 K. The hydrophobic SPION and the Wy5a-SPIO/Dtxl-NPs showed the coercivities of 200 Oe and 138 Oe, respectively. At room temperature, both of them turned into superparamagnetic, showing zero coercivity and remanence. There was no significant difference in the saturation magnetization between Wy5a-SPIO/Dtxl-NPs (63.04 Fe emu g^−1^) and hydrophobic SPION (65.16 Fe emu g^−1^), indicating negligible loss in magnetization per Fe unit when SPION were encapsulated into NPs. Therefore, the reserved superparamagnetic property of the SPION encapsulated in the nanoparticles was important for the NPs as an MRI-trackable drug delivery system.

The effect of SPION on spin-spin relaxation time (*T*_2_) shortening was produced by the susceptibility difference between the magnetic nanocrystals and surrounding medium, resulting in microscopic magnetic field inhomogeneity in the presence of an externally applied magnetic field [13]. It has been reported that SPION clustered within polymeric micelles or liposomes can increase the *T*_2_ relaxivity (*r*_2_) value of SPION [15]. To evaluate the MRI relaxivities of Wy5a-SPIO/Dtxl-NPs, the transversal relaxation times (*T*_2_) of the water protons of the aqueous solutions containing Wy5a-SPIO/Dtxl-NPs were evaluated using a clinical 3.0 T MRI scanner at room temperature. As shown in Figure 3(c), the MRI signal intensity was decreased with the increase in Wy5a-SPIO/Dtxl-NPs concentration. The *T*_2_ relaxivity value of Wy5a-SPIO/Dtxl-NPs was calculated as 188.59 mM^−1^·sec^−1^, which is significantly higher than commercially available hydrophilic SPION Resovit (82 Fe mM^−1^·sec^−1^) [16].

### 2.4. Cell-Specific Endocytosis and Lysosome Colocalization of Targeted NPs

**Figure 4:**
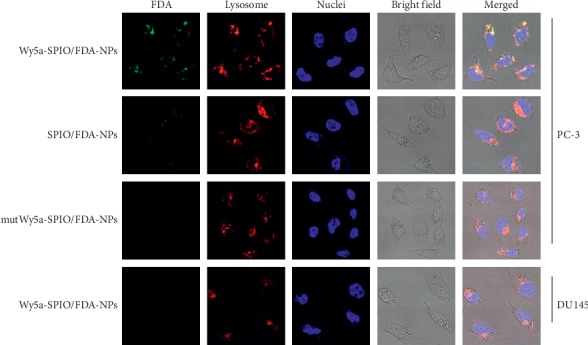
The uptake and intracellular distribution of Wy5a-SPIO/FDA-NPs in PC-3 cells by CLSM. The FDA dyes (green) were formulated into PLGA-PEG-aptamer triblock nanoparticles by nanoprecipitation instead of Dtxl. Nuclei and lysosomes were stained with Hoechst 33342 (blue) and Lyso-Tracker (Red), respectively.

The uptake and intracellular distribution of NPs were evaluated by laser confocal scanning microscopy (CLSM). PC-3 (Wy5a binding) and DU145 (Wy5a nonbinding) cells were utilized to examine whether Wy5a-SPIO/Dtxl-NPs were differentially endocytosed by PC-3 cells. To visualize the targeted nanoparticles by CLSM, FDA, a fluorescent dye, was encapsulated in the targeted nanoparticles instead of Dtxl. As shown in Figure 4, lysosome markers were colocalized with significant FDA fluorescence of Wy5a-SPIO/FDA-NPs in PC-3 cells, indicating a relatively rapid endocytosis of Wy5a-SPIO/FDA-NPs by PC-3 cells. Once inserted into cells, a majority of targeted nanoparticles were localized in the cytoplasmic compartments. In comparison, much less FDA fluorescence was observed in PC-3 cells after being incubated with mutWy5a-SPIO/FDA-NPs and nontargeted SPIO/FDA-NPs or in DU145 cells after being incubated with Wy5a-SPIO/FDA-NPs. These results suggested that a targeted delivery of Wy5a-SPIO/FDA-NPs to PC-3 cells and rapid Wy5a-mediated endocytosis can facilitate the internalization of Wy5a-encoded nanoparticles by PC-3 cells.

### 2.5. *In Vitro* Internalization Assay

**Figure 5:**
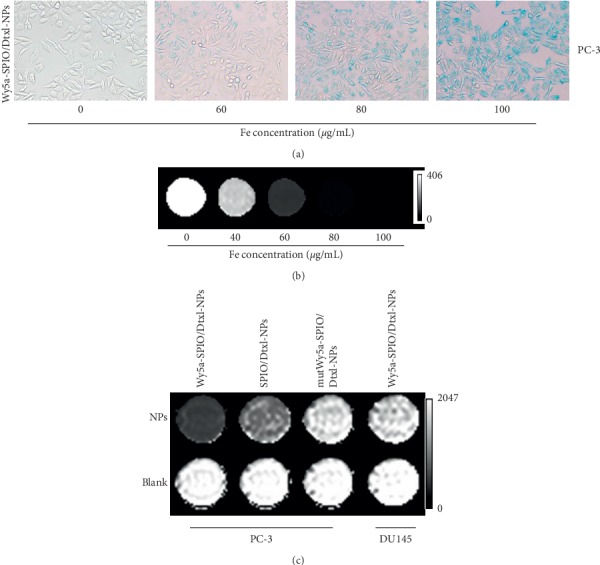
(a) Prussian blue staining images (×200) of PC-3 cells incubated with Wy5a-SPIO/Dtxl-NPs at Fe concentrations of 0, 60, 80, and 100 *μ*g/mL. (b) T2-weighted imaging of PC-3 cells incubated with Wy5a-SPIO/Dtxl-NPs at a series of Fe concentrations. (c) *In vitro* T2-weighted images of PC-3 cells incubated with Wy5a-SPIO/Dtxl-NPs, SPIO/Dtxl-NPs, and mutWy5a-SPIO/Dtxl-NPs and DU145 cells incubated with Wy5a-SPIO/Dtxl-NPs.

For the purpose of determining the nanoparticles internalization into cancer cells, the PC-3 cells incubated with a series of concentrations of Wy5a-SPIO/Dtxl-NPs were subject to Prussian blue staining. As illustrated in Figure 5(a), the intensity of Prussian blue staining became stronger over the increasing concentration of NPs. When the concentration of Fe in the culture media reached 60 *μ*g/mL, blue spots began to appear in the cytoplasm of PC-3 cells incubated with Wy5a-SPIO/Dtxl-NPs.

Similarly, the uptake of SPIO-loaded nanoparticles by targeted cells shortened the spin-spin relaxation time (*T*_2_) by dephasing the spins of neighboring water protons, leading to the darkening of *T*_2_-weighted images. As shown in Figure 5(b), PC-3 cells were incubated in a series concentrations of Wy5a-SPIO/Dtxl-NPs for 2 h, and the *T*_2_ images of cell suspension were obtained on a 3.0 T MRI scanner. The results showed that the *T*_2_ images of cell suspension became darker along with the increasing concentration of Fe. Consequently, the amount of SPIO-loaded nanoparticles internalized by the cells was increased corresponding to the concentration of NPs in the culture medium, resulting in the gradual enhancement of *T*_2_ relaxivities.

Visible evidence of the differential targeted internalization of Wy5a-targeting nanoparticles in cancer cells was shown in Figure 5(c). A decrease in MRI signal intensity was observed in cancer cells after being incubated with SPIO-loaded nanoparticles, suggesting that SPION encapsulated in nanoparticles after being absorbed by cancer cells maintained their original morphology inside the cells for a period of time. However, PC-3 cells incubated with Wy5a-SPIO/Dtxl-NPs yielded a significant negative contrast enhancement compared with SPIO/Dtxl-NPs and mutWy5a-SPIO/Dtxl-NPs. In contrast, DU145 cells incubated with Wy5a-SPIO/Dtxl-NPs exhibited a comparable decrease in MRI signal intensity relative to PC-3 cells incubated with mutWy5a-SPIO/Dtxl-NPs.

These results were consistent with CLSM, confirming the targeted uptake of Wy5a-targeting nanoparticles by Wy5a-binding cells.

### 2.6. *In Vitro* Cytotoxicity

**Figure 6:**
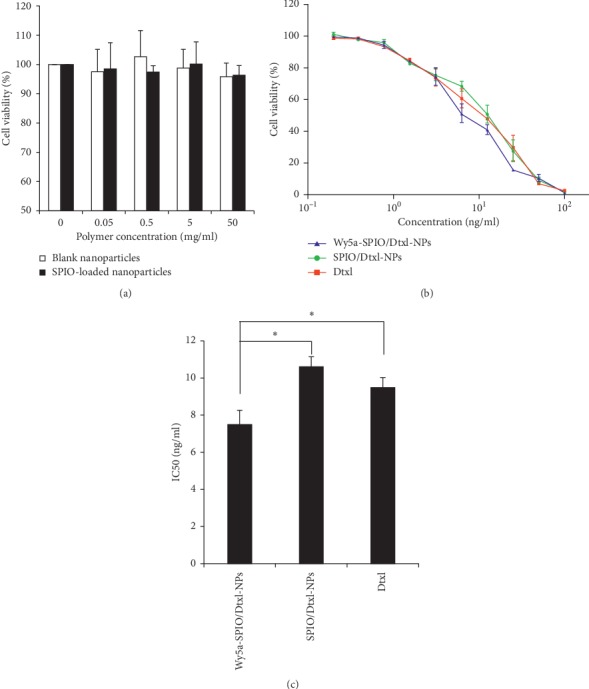
*In vitro* cytotoxicities of the blank nanoparticles, SPION-loaded nanoparticles, nontargeted nanoparticles, and targeted nanoparticles in PC-3 cells. (a) Cytotoxicities of the blank nanoparticles and SPION-loaded nanoparticles in PC-3 cells. (b) In vitro assessment of the antiproliferative effect of Wy5a-SPIO/Dtxl-NPs in PC-3 cells. (c) IC50 of Dtxl, SPIO/Dtxl-NPs, and Wy5a-SPIO/Dtxl-NPs in PC-3 cells. Data were detected by CCK-8 assay and presented as mean ± standard error (*n* = 3). ^*∗*^*P* < 0.05.

*In vitro* differential cytotoxicity of Wy5a-SPIO/Dtxl-NPs and nontargeted SPIO/Dtxl-NPs and Dtxl was comparatively analyzed using PC-3 cells. As illustrated in Figure 6(a), blank vesicles and SPIO-loaded nanoparticles showed no significant cytotoxicity in PC-3 cells even when the polymer concentration reached 50 mg/ml. Because Dtxl is a hydrophobic and membrane-permeable drug, the Dtxl cytotoxicity was evaluated due to the uptake of nanoparticles and subsequent intracellular drug release. The nanoparticles were incubated with PC-3 cells for 24 h to allow for the uptake of cells and subsequently incubated in nondrug medium for another 4 d before the measurement of cell viability. As shown in Figure 6(b), both Dtxl and Dtxl-encapsulated nanoparticles showed cytotoxicity on PC-3 cells in a sigmoidal dose-dependent manner. Our data showed that Wy5a-SPIO/Dtxl-NPs were significantly more cytotoxic as compared with nontargeted counterpart and Dtxl. The IC50 value of Wy5a-SPIO/Dtxl-NPs was 1.42-fold and 1.27-fold lower than those of nontargeted nanoparticles and Dtxl, respectively (Figure 6(c)). The stronger antitumor efficacy of targeted nanoparticles than nontargeted nanoparticles or Dtxl was considered to be correlated with the higher intracellular drug concentration, probably because the enhanced uptake of targeted nanoparticles by PC-3 cells and the rapid intracellular drug release triggered by the acidic environment inside lysosomes resulted in the disintegration of nanoparticles.

### 2.7. *In Vivo* Antitumor Efficacy

**Figure 7:**
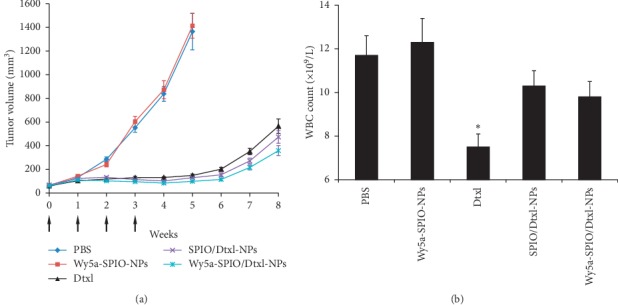
*In vivo* antitumor study on the xenograft nude mouse model of PCA. Mice treated with Wy5a-SPIO/Dtxl-NPs showed a superior antitumor efficacy and reduced drug toxicity. (a) Tumor volume in different groups. The arrows showed the time of injection. (b) White blood cell (WBC) count in different groups at the endpoint of observation. Data are expressed as mean ± standard error. ^*∗*^*P* < 0.05.

The therapeutic efficacy of the targeted nanoparticles *in vivo* was assessed using the xenograft models of PCA. As illustrated in Figure 7(a), none of the mice in the PBS and targeted SPIO-loaded nanoparticles (Wy5a-SPIO-NPs) groups exhibited tumor regression. The tumor growth accelerated and reached endpoint at week 5. The mice in the Dtxl, SPIO/Dtxl-NPs, and Wy5a-SPIO/Dtxl-NPs groups presented with initial tumor regression, while exhibiting tumor progression subsequently. However, Wy5a-SPIO/Dtxl-NPs could more effectively inhibit the tumor compared with nontargeted counterpart and Dtxl, especially after week 5. The *in vivo* toxicity was assessed by analyzing the effect on the WBC count at the endpoint of observation. As shown in Figure 7(b), the WBC count in Dtxl group was significantly lower than the other group. The white blood cell (WBC) count of Wy5a-SPIO/Dtxl-NPs and SPIO/Dtxl-NPs was within the normal range, indicating that nanoparticles could alleviate the Dtxl-induced toxicity. One reason for the enhanced efficacy and low toxicity of Wy5a-SPIO/Dtxl-NPs may be that the targeted nanoparticles were accumulated via passive tumor targeting effect after systemic administration and internalized into tumor cells via active targeting effect with subsequent intracellular release of Dtxl. Correspondingly, nontargeted SPIO/Dtxl-NPs could reach the tumor only by passive targeting. Moreover, the nanoparticles released significantly less Dtxl into the blood circulation and extracellular space than Dtxl before the nanoparticles reached the tumor, leading to the lower toxicity of nanoparticles.

## 3. Discussion

Cell type-specific delivery of cargoes is a critical goal for the applicability of nanotechnology in tumor therapy. Two approaches, active targeting and passive targeting, are commonly employed to target solid tumors [17]. For passive targeting, the nanoparticles have a higher probability to extravasate from the vascular compartment into tumor interstitium and to decrease the clearance of nanoparticles from the tumor interstitium due to the hypervascular permeability and impaired lymphatic drainage of tumor. For active targeting, the targeting moiety of nanoparticles specially recognizes the receptors expressed on the target cells and triggers receptor-mediated endocytosis. Subsequently, the nanoparticles are internalized into the targeted cells [17]. In the present study, multifunctional polymeric nanoparticles were synthesized for tumor-targeted intracellular drug release and MRI. The prostate cancer specific aptamer Wy5a was introduced on the nanoparticle surface as a targeting moiety, which has been developed for CRPC cell line PC-3 by cell-SELEX technique in our previous study [12]. Wy5a showed a high specificity to PC-3 cells rather than other cancer cells, such as DU145, 22RV-1, HeLa, and SMMC-7721 [12]. Our data showed that the therapeutic efficacy of different formulations was significantly correlated with their intracellular delivery efficiency. The aptamer Wy5a facilitated the intracellular delivery of polymeric nanoparticles. Therefore, targeted polymeric nanoparticles could more effectively suppress tumor growth than nontargeted nanoparticles *in vitro* and *in vivo*.

In our previous study, the formulation of targeted nanoparticles was involved with a series of chemical processes including the synthesis of drug-encapsulated nanoparticles, followed by surface functionalization, and conjugation of targeting moiety [13, 18]. However, the multistep synthesis process, which resulted in inherently inefficient systems, was likely to lead to batch-to-batch variations and possess limited ability to precisely engineer the NP surface properties [14]. In this study, the prefunctionalized biomaterials, which had all of the desired nanoparticle components, were developed before the formulation of nanoparticles. Furthermore, the prefunctionalized biomaterials were utilized to self-assemble the targeted nanoparticles in one step with a simple purification procedure, avoiding the need for subsequent postparticle modification. The quantity of aptamer on the surface of nanoparticles could be adjusted by changing the ratios of PLGA-b-PEG-b-Wy5a with PLGA-b-PEG to avoid the unnecessary masking of Apt on the nanoparticle surface, which was optimized to be maximally targeted and maximally stealth. In addition, biocompatible PEG and PLGA, FDA-approved polymers, are considered to be well tolerated for potential pharmaceutical applications.

Many potential therapeutic applications of nanoparticles in tumor therapy require systematic administration. Targeted nanoparticles allow the preferential delivery of therapeutic agents to the intended site. Once the nanoparticles were internalized into lysosomes by tumor cells, this could result in the disintegration of nanoparticles and subsequent rapid drug release [19]. However, before the nanoparticles were internalized by targeted cells, the nanoparticles or Dtxl dissociated from nanoparticles was inevitably absorbed by other organs and cells like the reticuloendothelial systems, leading to nontargeted toxicity and side effect. Therefore, reducing nontargeted endocytosis and/or the release of Dtxl from the nanoparticles before the nanoparticles reach the target site could mitigate the side effect [20]. In our formulation, hydrophilic PEG blocks formed a corona-like shell to reduce the systemic clearance and prolong circulation half-life to allow the nanoparticle to reach the target site. Moreover, our previous study demonstrated that introduction of hydrophobic SPION into targeted nanoparticles can facilitate the controlled drug release [13]. Consequently, targeted nanoparticles in this study showed controlled drug release for more than 5 d and less Dtxl-induced toxicity *in vivo*.

Currently, gadolinium chelate contrast-enhanced MRI is one of the primary methods for the diagnosis of prostate cancer due to its high spatial resolution and noninvasive nature. Unfortunately, gadolinium chelates is a nontargeted contrast agent and likely to diffuse from tumor site. Over the past few years, SPIO-based nanoparticles have been applied in clinical practice as MRI contrast agents, such as Resovit, Endorem, and Feridex IV [21]. However, these SPIO-based nanoparticles depended on passive targeting rather than targeted tumor cell labeling. Therefore, these systems have no significant advantages over the gadolinium chelates [21]. The next-generation tumor-targeted contrast agents are expected to improve the diagnosis of tumors due to enhanced cellular internalization and slower clearance from the tumor site. Moreover, the nanomedical platforms can make the nanoparticles visible by simultaneously incorporating the therapeutic and imaging agent [22]. In this study, MRI-visible polymeric nanoparticles with multifunctional tumor-targeted imaging and therapy were investigated. Our results indicated that multifunctional polymeric nanoparticles reserved superparamagnetic property and saturation magnetization of SPION. Meantime, the clustering of SPION inside nanoparticle core resulted in an increased *T*_2_ relaxivity value compared to commercially available Resovit. The internalization of SPIO-loaded polymeric nanoparticles by the tumor cells induced significant T2-contrast enhancement detected by MRI.

## 4. Conclusion

In summary, multifunctional targeted nanoparticles simultaneously encapsulating SPION and Dtxl were successfully developed based on a prefunctionalized, well-defined triblock copolymer (PLGA-b-PEG-b-Wy5a) via self-assembly method. The targeted NPs exhibited the differential delivery to CRPC cells and enhanced relaxivities of MRI by the cluster of SPION in the NPs and controlled drug release, resulting in increased antitumor efficacy and in vitro MRI contrast enhancement, indicating that the multifunctional targeted nanoparticles have a great potential for CRPC therapy.

## 5. Materials

Carboxyl-modified poly(D,L-lactic-co-glycolic acid) with a 50 : 50 monomer ratio and viscosity of 0.65–0.85 dl/g was purchased from Absorbable Polymers International (Pelham, AL). Heterofunctional poly(ethylene glycol) (PEG) terminated by carboxylic acid and amine (NH_2_-PEG-COOH, 3.4 kDa) were obtained from Peking JenKem Technology Co., Ltd. (Beijing, China). Fluorescein diacetate (FDA), N-hydroxysuccinimide (NHS), and 1-ethyl-3-(3-dimethylaminopropyl) carbodiimide hydrochloride (EDC·HCl) were purchased from Sigma-Aldrich. Dialysis tubes (MWCO: 1, 3.5, 7, 10 kDa) were purchased from Shanghai Green Bird Technology Development Co., Ltd., China, and were stored in 1 mM ethylene diamine tetraacetic acid (EDTA) aqueous solution prior to use. Cell culture medium (RPMI 1640), trypsin-EDTA, and fetal bovine serum (FBS) were purchased from Invitrogen Corporation (Carlsbad, CA, USA). Cell lines (PC-3 and DU145) were purchased from American Type Culture Collection (ATCC) (Manassas, VA). The passage of the cell line used for the inoculation was less than 8. All solvents were of analytical grade. Hydrophobic SPION (6 nm) were synthesized using a previously described method [15].

### 5.1. Synthesis of PLGA-B-PEG3.4k-b-Wy5a Triblock Copolymer

Carboxylate-functionalized copolymer PLGA-b-PEG3.4k-COOH was synthesized by the conjugation of COOH-PEG3.4k-NH_2_ to PLGA-COOH as described previously. [23] The single strand DNA Apt Wy5a (sequence: 5′-NH2-spacer-TGCCACTACAGCTGGTTCGGTTTGGTGACTTCGTTCTTCGTTGTGGTGCTTAGTGGC) with a 5′-amino group attached by a hexaethylene glycol spacer was synthesized as described previously [12]. To synthesize PLGA-b-PEG3.4k-b-Wy5a, PLGA-b-PEG3.4k-COOH was activated with 1-ethyl-3-[3-dimethylaminopropyl]carbodiimide hydrochloride (EDC) and N-hydroxysuccinimide (NHS) in methylene chloride, and the resulting PLGA-b-PEG3.4k-NHS was washed by cold methanol, dried, and resolubilized in acetonitrile. The DNA Apt Wy5a was dissolved in a mixture of formamide/acetonitril and then reacted with PLGA-b-PEG3.4k-NHS at room temperature. The resulting PLGA-b-PEG3.4k-b-Wy5a was dialyzed with cold methanol and dried in vacuum for NP preparation. PLGA-b-PEG3.4k-b-mutWy5a was synthesized by the same method described above using mutWy5a (5′-NH2-spacer TGCCACTACAGCTACCCCTTTAATCCCAAACCCTTCTTCGTTGTGGTGCTTAGTGGC) instead of Wy5a.

### 5.2. Preparation and Characterization of Nanoparticles

NPs were prepared by using a nanoprecipitation method. In brief, a mixture of PLGA-b-PEG3.4k-b-Wy5a/PLGA-b-PEG3.4k (20 mg/ml, molar ratio, 1 : 4), Dtxl (2 mg/ml), and SPIO (2 mg/ml) was dissolved in acetonitrile/formamide (60 : 40 vol/vol), mixed with sonication (60 Sonic Dismembrator, Fisher Scientific), dropwise added to water, and adjusted to the final NP concentration of 3 mg/ml. The resulting NPs were allowed to self-assemble for 2 h with continuous stirring at room temperature, while the organic solvent was allowed to evaporate. The remaining organic solvent and free molecules were washed by an Amicon centrifugation filtration membrane with a molecular mass cutoff of 10 kDa. NPs were lyophilized to obtain the powdery form in order to avoid Dtxl release for subsequent experiments. FDA-loaded NPs were prepared by the same method using FDA instead of Dtxl. The aptamers on the surface of NPs were determined by hydrolyzing the aptamers from the NP surface using 0.01 M Tris buffer at pH 9.5 at 40°C. Afterwards, aptamers were separated from nanoparticle debris by centrifugation by 16,000 *g* for 30 min. After centrifugation, the supernatant was collected and the concentration of aptamers was measured by measuring UV absorbance at a wavelength of 260 nm. The NP size and zeta potential were determined using a Zeta PALS dynamic light-scattering detector (15 mW laser, incident beam 676 nm, Brookhaven Instruments, Holtsville, NY). The NP morphology was characterized using TEM with a JEOL JEM-200CX instrument at an acceleration voltage of 200 kV.

### 5.3. Dtxl-Loaded and SPIO-Loaded Contents

The Dtxl-loaded content, defined as the weight percentage of Dtxl in the freeze-dried sample, was quantified by high-pressure liquid chromatography (Agilent 1100, Palo Alto, CA) equipped with a pentafluorophenyl column (Curosil-PFP, 250 × 4.6 mm, 5 *μ*m, Phenomenex, Torrance, CA) using a previously described method [13]. The absorbance of Dtxl at a wavelength of 227 nm was measured to determine the Dtxl concentration in the solution using a preestablished calibration curve. The SPIO-loaded content, defined as the weight percentage of SPIO in the freeze-dried sample, was quantified by Atomic Absorption Spectrophotometry (AAS) (Z-2000, Hitachi, Japan) as previously described [13]. The dried sample was weighed and redissolved in HCl solution (1 mol/L) before analysis. The absorbance of Fe was measured to determine the Fe concentration in the solution using a preestablished calibration curve.

### 5.4. *In Vitro* Dtxl Release Study

Dtxl release kinetics of NPs were determined using a previously described method [18]. In brief, Dtxl-loaded NPs were aliquoted into a series of semipermeable dialysis, mini-dialysis tubes (100 *μ*L in volume, molecular mass cutoff 10 kD, Pierce) and were then sealed in 40 L PBS to mimic the infinite sink condition. At a predetermined time, a fraction of NP sample was collected from the dialysis tubes, and the amount of Dtxl that remained in the NPs was measured by HPLC described above.

### 5.5. Magnetic Properties of NPs

The magnetization data of hydrophobic SPION and Dtxl/SPIO-loaded NPs were determined using a MPMS XL-7 Quantum Design SQUID magnetometer at 10 K and 300 K. Temperature control was achieved by the components within the Temperature Control Module (TCM) under the active control of the Model 1822 Controller and the control system software. The applied magnetic field was varied from 2 × 10^4^ Oe to −2 × 10^4^ Oe in order to generate hysteresis loops.

### 5.6. Prussian Blue Staining

Human prostate cancer PC-3 cells were seeded in 96-well plates at a density of 5 × 10^3^ cells per well and then incubated for 2 h with Dtxl/SPIO-loaded NPs in a RPMI 1640 medium supplemented with 10% FBS at 37°C. Afterwards, the cells were washed with PBS (pH 7.4), fixed with 4% glutaraldehyde for 10 min, and then incubated for 30 min at 37°C with 100 *μ*L Prussian blue solution containing 1% hydrochloride and 1% potassium ferrocyanide (II) trihydrate. The iron staining was observed using a microscope. DU145 cells, which aptamer Wy5a did not bind to, were used as the control cells.

### 5.7. Confocal Laser Scanning Microscopy (CLSM)

PC-3 cells were seeded in Petri dishes at a density of 5 × 10^3^ cells per dish and incubated in the presence of FDA/SPIO-loaded NPs in a RPMI 1640 medium supplemented with 10% FBS at 37°C for 0.5 h. Afterwards, the cells were washed with PBS (pH 7.4) for microscopic observation on a CLSM FV1000 (OLYMPUS, 40 Japan) with a confocal plane of 300 nm. To identify the nanoparticles location inside cells, nuclei were stained blue with Hoechst 33342 (Molecular Probes), and the lysosomes were stained red with Lyso-Tracker-Red (Molecular Probes). Hoechst 33342, FDA, and Lyso-Tracker-Red were excited at 352, 450, and 577 nm with emissions at 455, 520, and 590 nm, respectively. DU145 cells were used as the control cells.

### 5.8. *In Vitro* MRI

MRI measurements were performed on a clinical 3.0 T MRI scanner (Philips Intera, Netherland, B. V.) at room temperature. A 6-cm animal coil (Chenguang Medical Technologies, Shanghai, China) was used for all *in vitro* MRI studies. The nanoparticles or cancer cells after being incubated with SPION-loaded nanoparticles for 2 h were resuspended in the solution containing 2% gelatin (Sigma-Aldrich (Shanghai) Trading, China). *T*_2_-weighted images were acquired using the following parameters: TR/TE, 2600/100 ms; FOV, 40 mm × 60 mm; matrix, 256 × 384; section thickness, 2 mm. *T*_2_ relaxation data were acquired by using a single-section mixed inversion-recovery spin-echo sequence that was initiated with an inversion-recovery pulse (2000/160 and inversion time msec, 400) followed by a spin-echo pulse (4000/20) and a single-section multi-spin-echo (2000/160). Both sequences were performed with the following parameters: stepped echo time, 20–160 msec for 8 eight steps; echo spacing, 20 msec; FOV, 40 mm × 60 mm; matrix, 256 × 384; section thickness, 2 mm. In both cases, a circular region of interest was selected in each sample, and the values of *T*_2_ relaxation times were obtained. The increase in *r*2 relaxation rates (1/*T*) with increasing Fe concentration was analyzed by linear least squares regression analysis. *T*_2_ relaxivities were calculated from the slope of the linear plots of *r*_2_ relaxation rates versus Fe concentration.

### 5.9. *In Vitro* Cytotoxicity

Cytotoxicities of blank NPs, Dtxl, SPION-loaded NPs, and nontargeted and targeted Dtxl/SPIO-loaded NPs against PC-3 cells were measured by cell counting kit-8 assay (CCK-8, Dojindo Laboratories, Japan). Experiments were conducted in triplicate. The cells were dispensed in 96-well plates at a density of 4 × 10^3^ cells per well, maintained in 100 *μ*L of RPMI 1640 medium supplemented with 10% FBS, and incubated for 24 h at 37°C in a humidified atmosphere with 5% CO_2_. Then, Dtxl and NPs at different drug concentrations were added to cell culture medium. After 24-h incubation, the medium was replaced and no further dose of drug was added. After 4-d incubation, 10 *μ*L of CCK-8 solution was added to each well and incubated with the cells for 1 h. The absorbance at 450 nm of each well was recorded on a microplate reader (Molecular Devices, USA). IC50 values were quantitatively calculated by GraphPad Prism software (San Diego, CA) using nonlinear regression analysis.

### 5.10. *In Vivo* Antitumor Efficacy

Animal studies were conducted in accordance with the guidelines of the Institutional Animal Care and Use Committee at Sun Yat-sen University. Male severe combined immunodeficiency (SCID) mice, aged 5–7 weeks, were inoculated subcutaneously with 5 × 10^5^ PC-3 cells. Treatment was started when the tumors reached approximately 50 mm^3^ in volume. Mice were randomly divided into five groups of 10 mice per group: (1) PBS; (2) Wy5a-SPIO-NPs (without Dtxl); (3) Dtxl (10 mg/kg); (4) Dtxl/SPIO-NPs (10 mg/kg Dtxl); (5) Wy5a-Dtxl/SPIO-NPs (10 mg/kg Dtxl). Treatment was administered once a week for 4 weeks by tail vein injection. Body weight and tumor size of the mice were monitored once a week for 8 weeks. Tumor volume was calculated using the following formula: length × width × height × 0.5236. If body weight loss persisted beyond 20% of predose weight or the tumor size exceeded 1500 mm^3^, the animals were euthanized. The tumor size at the time of euthanasia was used to calculate the mean tumor size. When mice were euthanized, 1 ml of blood was drawn through cardiac puncture and analyzed for a toxicity profile of the treatment regimens.

## Data Availability

The data used to support the findings of this study are available from the corresponding author upon request.
